# A machine-learning model for staging autoimmune gastritis based on endoscopic and serological features

**DOI:** 10.3389/fimmu.2026.1883686

**Published:** 2026-07-29

**Authors:** Jingna Tao, Linghan Meng, Tong Li, Jiaqi Wang, Liju Zhang, Xiyan Zhang, Zhihong Li

**Affiliations:** 1 Department of Gastroenterology, Dongzhimen Hospital, Beijing University of Chinese Medicine, Beijing, China; 2 Guang’anmen Hospital, China Academy of Chinese Medical Sciences, Beijing, China

**Keywords:** autoantibodies, Barrett esophagus, endoscopy, gastrointestinal, gastritis, atrophic, machine learning

## Abstract

**Objectives:**

Autoimmune gastritis (AIG) lacks a unified, practical staging system. The AIG-atrophic stage (AIG-AS) scheme, based on the proportional area of remnant oxyntic mucosa (ROM), is promising but observer-dependent. We developed machine-learning models to stage AIG using endoscopic and serological features.

**Methods:**

This single-center cross-sectional study enrolled 203 patients with AIG confirmed by integrated endoscopic, histological, and serological criteria between December 2023 and December 2025. White-light endoscopy (WLE), magnifying endoscopy with narrow-band imaging (ME-NBI), and a serum panel were collected. Patients were classified under AIG-AS as Stage 1 (50%<ROM ≤ 100%), Stage 2 (10%<ROM ≤ 50%), or Stage 3 (ROM ≤ 10%), and additionally as early-to-intermediate (Stages 1 + 2) versus advanced (Stage 3). Seven algorithms—LASSO logistic regression, elastic net, random forest, XGBoost, support vector machine, gradient boosting, and stacking—were trained on five feature sets using stratified 5-fold nested cross-validation, with SMOTE applied only within training folds. SHAP analysis provided interpretability.

**Results:**

Stages 1, 2, and 3 included 87 (42.9%), 36 (17.7%), and 80 (39.4%) patients. The combined endoscopy+serology random forest achieved the highest three-class macro-AUC (0.878 ± 0.051; accuracy 74.9%), surpassing WLE stacking (0.853 ± 0.056) and serology-only models (≤0.693). For binary staging, the WLE+ME-NBI random forest reached an AUC of 0.913 ± 0.046. Loss of gastric folds, gastrin-17, pepsinogen I, crypt-opening grade, and cast-off skin appearance were the dominant predictors. Barrett esophagus prevalence was 66.8%, with long-segment Barrett esophagus 3.3-fold more common in advanced AIG than in earlier disease.

**Conclusions:**

Multi-modal machine learning enables accurate, interpretable AIG staging. Endoscopic features outperform serology, and combined models offer the best three-class performance. The high prevalence of Barrett esophagus supports routine esophageal surveillance in AIG.

## Introduction

Autoimmune gastritis (AIG) is a chronic, organ-specific autoimmune disease characterized by T-cell–mediated destruction of gastric parietal cells, achlorhydria, and progressive corpus-fundic atrophy (1–3). It predominantly affects middle-aged and older women and is frequently associated with other autoimmune conditions, including autoimmune thyroid disease and type 1 diabetes (1). Because the clinical presentation is insidious, a substantial proportion of patients are diagnosed only after the disease has reached an advanced stage.

AIG evolves continuously from early inflammatory infiltration, through florid replacement of oxyntic glands by pseudopyloric metaplasia, to advanced glandular remodeling dominated by intestinal metaplasia, with stage-specific endoscopic, serological, and clinical risk profiles (2). In advanced disease, persistent hypergastrinemia and enterochromaffin-like cell hyperplasia confer a markedly elevated risk of type 1 gastric neuroendocrine tumor and require structured endoscopic surveillance; in earlier stages, autoimmune injury remains active and may, in principle, be modifiable (4). Accurate within-disease staging is therefore a prerequisite for individualized surveillance and risk stratification.

Despite this clinical importance, no unified staging system for AIG exists. The Kimura–Takemoto classification (5) was developed for the antrum-to-corpus progression of *Helicobacter pylori*–associated atrophic gastritis and does not fit the “reverse atrophy” of AIG (2). Histological three-stage classifications (6) comprehensively capture progression but rely on targeted biopsy. Because AIG atrophy is spatially non-uniform, point sampling poorly represents the mucosa as a whole, and the diffusely thinned wall raises the bleeding risk of multi-site biopsy. In one histologically confirmed cohort, every patient was classified as active or end-stage, suggesting a substantial blind spot for early disease (7). A central unmet need is therefore an objective, “whole-field” endoscopic assessment of atrophic progression that draws on overall mucosal features.

Maruyama and colleagues recently proposed an AIG-atrophic stage (AIG-AS) scheme based on the proportional area of remnant oxyntic mucosa (ROM): Stage 1 (50%<ROM ≤ 100%), Stage 2 (10%<ROM ≤ 50%), and Stage 3 (ROM ≤ 10%) (8). ROM provides a whole-mucosa, dynamic estimate of residual oxyntic gland (2). However, ROM area is judged subjectively and observers frequently disagree near stage cut-offs, with a moderate inter-observer Kappa of about 0.50 reported in multicenter validation (9). Building objective, quantitative, multi-dimensional predictors on top of AIG-AS therefore remains an open problem.

Machine learning has been successfully applied to OLGA/OLGIM staging of *H. pylori*–associated atrophic gastritis (10) and to image-based AIG diagnosis (11, 12), with multicenter deep-learning systems reaching AUCs above 0.97. To our knowledge, however, no study has applied machine learning to within-AIG stage prediction. We therefore enrolled patients with integrated-criteria AIG diagnoses, collected WLE features, ME-NBI features, and a serological panel, adopted AIG-AS as the staging framework, and built a multi-algorithm pipeline to construct stage-prediction models and quantify each feature’s contribution. The aim was to provide an evidence base for precision AIG staging and individualized surveillance.

## Methods

### Study design and patients

This single-center, cross-sectional study enrolled patients diagnosed with AIG at the Department of Gastroenterology, Dongzhimen Hospital, Beijing University of Chinese Medicine, between December 2023 and December 2025. All patients underwent standard upper gastrointestinal endoscopy with conventional WLE; a subset additionally underwent ME-NBI on the same examination. Serum assays were obtained around the time of endoscopy. Of 235 initially eligible patients, 32 were excluded, leaving 203 patients and 11,557 endoscopic images for analysis. Endoscopic examinations were performed by attending-level or more senior gastroenterologists with at least five years of experience. The study was approved by the Ethics Committee of Dongzhimen Hospital (approval no. DZM-EC-2025-540) and conducted in accordance with the Declaration of Helsinki. This was a retrospective cross-sectional study using de-identified clinical data and archived endoscopic images; the requirement for written informed consent was therefore waived by the Ethics Committee.

### Diagnostic and staging criteria

AIG was confirmed by integrated endoscopic, histological, and serological criteria following the 2023 Japanese diagnostic standard for type-A gastritis (13) and the 2024 *Stomach and Intestine* monograph (2, 3, 8). Inclusion required: (i) corpus-fundic atrophy on endoscopy and/or biopsy; (ii) positive parietal cell antibody (PCA) and/or intrinsic factor antibody; (iii) negative ^13^C/^14^C urea breath test or rapid urease test; (iv) age 18–85 years; (v) complete endoscopic image records. Exclusion criteria were previous gastric surgery, histologic malignancy other than gastric neuroendocrine tumor, atrophy of non-autoimmune etiology, severe comorbidity, pregnancy or lactation, and incomplete records. AIG stage was assigned under AIG-AS (8). A binary scheme combined Stages 1–2 into early-to-intermediate AIG and retained Stage 3 as advanced, mirroring the clinical question of when to initiate intensified neoplastic surveillance.

### Feature collection

WLE features of the corpus, fundus, antrum, incisura angularis, and esophagus were recorded, including mucosal thinning, visible submucosal vessels, loss of gastric folds, swelling of the areae gastricae, mucosal edema, redness patterns (spotty, map-like, diffuse), sticky adherent dense mucus, hyperplastic polyps, ROM morphologic subtype, preserved antral mucosal extent (Grades 0–3), and Barrett esophagus (BE) grade (none/short-segment <3 cm/long-segment ≥3 cm) by Prague C&M criteria (14). ME-NBI features included pseudopyloric metaplasia, cast-off skin appearance (CSA), white globe appearance (WGA) (15), marginal-crypt-epithelium (MCE) swelling, white opaque substance, light blue crest, demarcation line, irregular microvascular/microsurface patterns, and crypt-opening (CO) grade (1, pinpoint; 2, blurred; 3, short rod-shaped; 4, absent) (16). Serum indicators comprised pepsinogen I (PGI), pepsinogen II (PGII), the PGI/PGII ratio, gastrin-17 (G-17), PCA titer (1:40 to 1:3200; log-transformed and treated as ordinal), folate, vitamin B12, hemoglobin, and thyroid autoantibodies (TPOAb, TgAb, TRAb). Indicators with >50% missingness (anti–*H. pylori* type I/II antibodies, intrinsic factor antibody, thyroid function tests) were excluded from modeling. Image interpretation was performed independently by two senior endoscopists with adjudication by a third in case of disagreement; histology was independently reviewed by two pathologists.

### Statistical analysis

Continuous variables are reported as mean ± SD or median (Q1–Q3); categorical variables as n (%). Three-group comparisons used one-way ANOVA or Kruskal–Wallis tests for continuous variables and χ² or G-tests for categorical variables; binary comparisons used independent t-tests or Mann–Whitney U tests, and χ²/Fisher exact tests. Bonferroni-corrected pairwise tests followed significant overall differences. Effect sizes were η²/ϵ² (continuous, three-group), Cramér V (categorical), Cohen d (continuous, binary), or rank-biserial r. Two-sided α=0.05. Analyses used SPSS 30.0 (IBM, Armonk, NY, USA).

### Machine-learning modeling

Five feature sets were constructed: (i) WLE (n=203); (ii) WLE+ME-NBI (n=160); (iii) core serum (7 indicators with missingness <50%: PGI, PGII, PGI/PGII ratio, PCA titer, G-17, folate, vitamin B12); (iv) extended serum (11 indicators, adding TPOAb, TgAb, TRAb, hemoglobin); (v) combined endoscopy+serology (WLE + core serum, 31 variables). Each set was modeled with seven algorithms—LASSO logistic regression, elastic net, random forest, XGBoost, support vector machine, gradient boosting, and a stacking ensemble (random forest, XGBoost, and SVM base learners, logistic regression meta-learner). Stratified 5-fold cross-validation was used, with nested 3-fold randomized search (ten candidate combinations per model) for hyperparameter tuning inside each training fold. Preprocessing—per-feature median imputation, zero-variance removal, Z-score standardization—and SMOTE for class imbalance (Stage 2, n=36) were applied only inside training folds to prevent information leakage; test folds retained the original class distribution. The primary metric was macro-averaged AUC for the three-class task and AUC for the binary task. Feature importance was extracted with Gini importance (tree models), standardized coefficients (linear models), or permutation importance (SVM and stacking); the best tree-based model per set was further analyzed with SHAP. Multicollinearity in the combined set was assessed with Pearson correlation (|r|>0.7) and the variance inflation factor (VIF>10). Implementation used Python 3.13 with scikit-learn, XGBoost, imbalanced-learn, and SHAP.

## Results

### Patient characteristics

**Figure 1:**
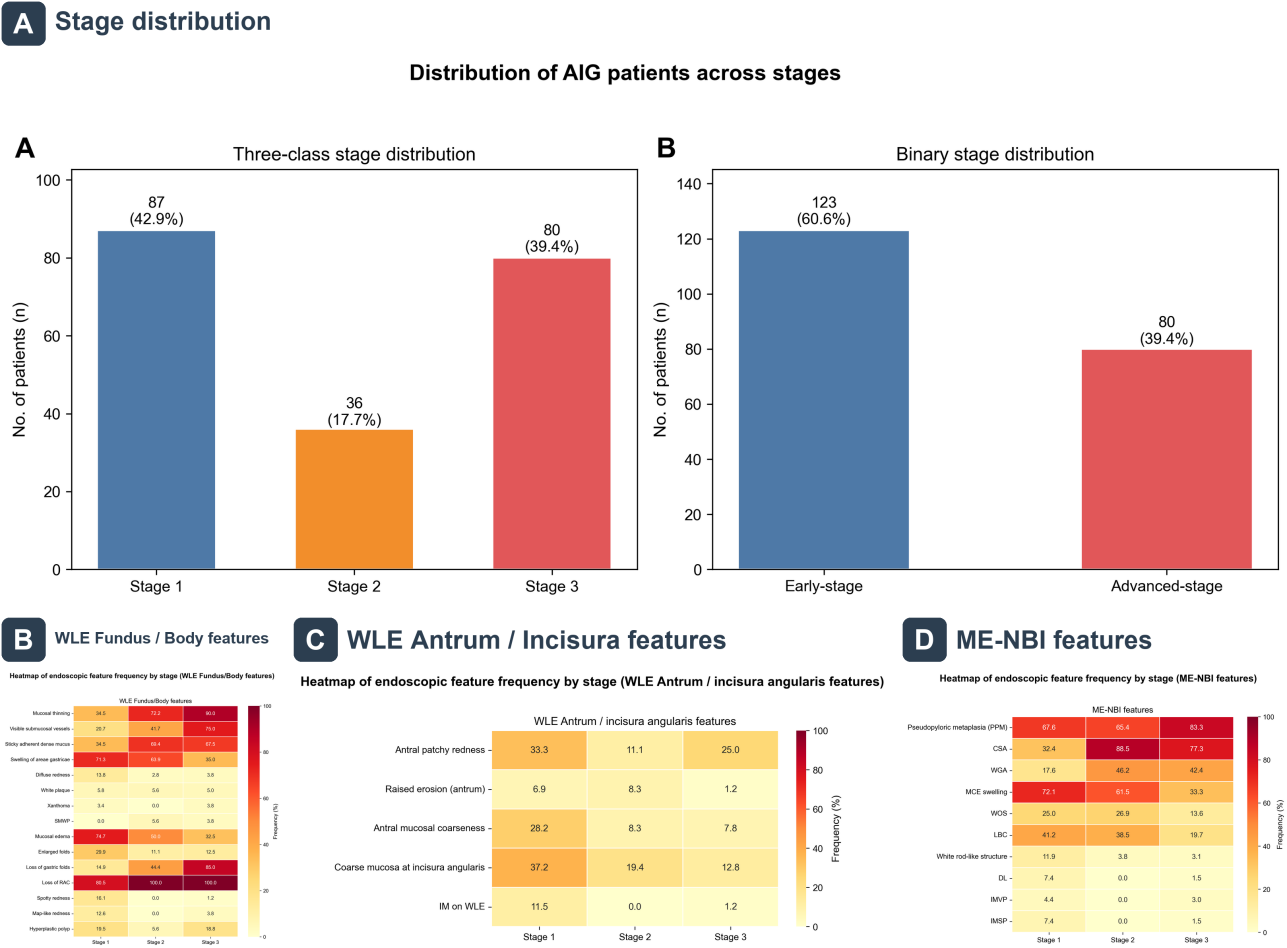
Stage distribution and frequency of endoscopic features across AIG-AS stages. **(A)** Distribution of patients across the three-class and binary schemes. **(B)** Heatmap of WLE feature frequency across stages — corpus and fundus. **(C)** Heatmap of WLE feature frequency — antrum and incisura angularis. **(D)** Heatmap of ME-NBI feature frequency across stages.

Of 203 patients, 131 (64.5%) were women and 72 (35.5%) men, a 1:1.8 ratio; the median age was 62 years (Q1–Q3, 54–67). The AIG-AS distribution was 87 (42.9%) Stage 1, 36 (17.7%) Stage 2, and 80 (39.4%) Stage 3; the binary distribution was 123 (60.6%) early-to-intermediate and 80 (39.4%) advanced. Age and sex did not differ significantly across stages (P>0.05) (**Table 1**; **Figure 1A**). One hundred and sixty patients additionally underwent ME-NBI (68 Stage 1, 26 Stage 2, 66 Stage 3).

**Table 1:** AIG-AS staging based on the proportional area of remnant oxyntic mucosa (ROM) and corresponding endoscopic findings.

AIG-AS	ROM area	Endoscopic findings
Stage 1 (early)	50% < ROM ≤ 100%	Redness and swelling of the areae gastricae (granular, mosaic, or salmon-roe appearance)
Stage 2 (florid)	10% < ROM ≤ 50%	ROM of varied morphology and size; medium-to-large hyperplastic polyps; white globe appearance
Stage 3 (advanced)	ROM ≤ 10%	Marked atrophy with prominent visible submucosal vessels; sticky adherent dense mucus; white globe appearance; small hyperplastic polyps

### Endoscopic features across stages

On WLE, two oppositely directed feature clusters defined the staging spectrum (**Figures 1B, C**). Atrophy-related features increased monotonically with stage: mucosal thinning rose from 34.5% (Stage 1) to 90.0% (Stage 3); visible submucosal vessels from 20.7% to 75.0%; and loss of gastric folds from 14.9% to 85.0% (all P<0.001). Loss of the regular arrangement of collecting venules reached 100% at Stages 2–3 (P<0.001). Sticky adherent dense mucus was more frequent at Stages 2–3 (69.4% and 67.5%) than at Stage 1 (34.5%) (P<0.001). Conversely, inflammation- and edema-related features decreased with stage: mucosal edema fell from 74.7% to 32.5% (P<0.001), swelling of the areae gastricae from 71.3% to 35.0% (P<0.001), and enlarged folds from 29.9% to 12.5% (P<0.01). Redness features (spotty, map-like, diffuse) were most common at Stage 1 and largely absent thereafter (all P<0.05). Bonferroni-corrected pairwise tests confirmed that loss of gastric folds discriminated every stage transition (Stage 1 vs Stage 2, P = 0.003; Stage 2 vs Stage 3, P<0.001).

Among 160 patients with ME-NBI (**Figure 1D**), CSA rose sharply from 32.4% (Stage 1) to 88.5% (Stage 2) and 77.3% (Stage 3) (P<0.001), and WGA followed a similar pattern (17.6%, 46.2%, 42.4%; P = 0.002); MCE swelling decreased with stage (72.1%, 61.5%, 33.3%; P<0.001), mirroring the WLE pattern of areae-gastricae swelling. CO Grade 4 (absent openings) predominated at Stages 2–3 (73.1% and 87.3% vs 25.4% at Stage 1; P<0.001).

Barrett esophagus was strikingly prevalent: overall 66.8% (short-segment 41.3%; long-segment 25.5%). Long-segment Barrett esophagus rose from 13.4% (Stage 1) to 43.0% (Stage 3) (P<0.001), whereas short-segment Barrett esophagus was most common at Stage 1 (54.9%).

### Serological features

PGI fell progressively across stages (medians 67.6 → 30.2 → 23.7 μg/L; P<0.001), as did the PGI/PGII ratio (4.21 → 1.89 → 2.10; P<0.001). PGII decreased modestly (P = 0.002). G-17 followed a non-linear trajectory, peaking at Stage 2 (median 34.8 pmol/L) and lowest at Stage 1 (7.4 pmol/L) (P<0.001). Vitamin B12 declined progressively (581.5 → 411.0 → 375.0 pg/mL; P = 0.010). PCA positivity rose from 61.7% (Stage 1) to 85.3% (Stage 2) and 81.8% (Stage 3) (P = 0.005), while PCA titer did not differ across stages. TgAb was highest at Stage 3 (P<0.05); TPOAb, TRAb, folate, and hemoglobin did not differ significantly. Pairwise tests showed that PGI and the PGI/PGII ratio discriminated Stage 1 from Stages 2 and 3 but not Stage 2 from Stage 3—an intrinsic ceiling on serological staging in advanced disease.

### Machine-learning performance

**Figure 2:**
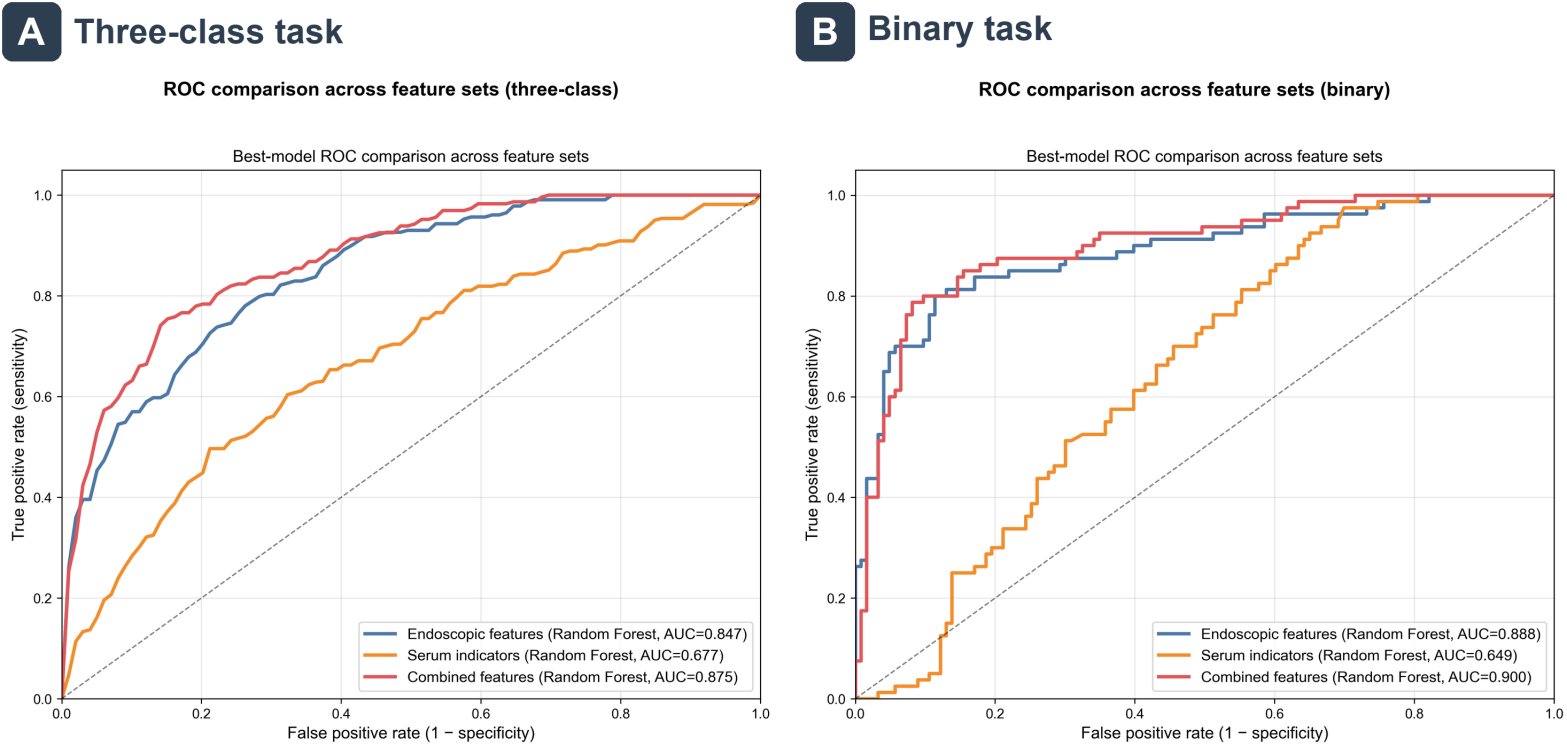
ROC curves comparing the best models across the five feature sets. **(A)** Three-class task (macro-averaged). **(B)** Binary task (early-to-intermediate vs advanced).

Best-model performance per feature set is shown in **Table 2**. For the three-class task, the combined endoscopy+serology random forest achieved the highest macro-AUC (0.878 ± 0.051) and accuracy (74.9%), followed by the WLE stacking ensemble (0.853 ± 0.056) and the WLE+ME-NBI elastic net (0.841 ± 0.038). Serology-only models lagged substantially (core stacking 0.693 ± 0.075; extended random forest 0.659 ± 0.051). For the binary task, the WLE+ME-NBI random forest achieved the highest AUC (0.913 ± 0.046), with sensitivity 0.816 ± 0.089 and specificity 0.827 ± 0.134; the combined random forest reached AUC 0.907 ± 0.065 with the highest specificity (0.886 ± 0.033) and accuracy (85.2%); the WLE-only random forest achieved AUC 0.887 ± 0.068. Both serology-based binary models remained ≤0.693. Overlaid ROC curves (**Figure 2**) showed the combined and endoscopy-based curves consistently above the serology curves; confusion-matrix analysis indicated that Stage 1 and Stage 3 were correctly identified in over 80% of cases by the best models, whereas Stage 2 was most often misclassified into the adjacent stages.

**Table 2:** Best-model performance per feature set in three-class and binary AIG staging tasks.

Feature set	Best model	Three-class macro-AUC	Three-class accuracy	Binary AUC	Binary specificity
Endoscopy (WLE)	Stacking/RF	0.853 ± 0.056	0.680 ± 0.079	0.887 ± 0.068	0.886 ± 0.017
Endoscopy (WLE+ME-NBI)	Elastic net/RF	0.841 ± 0.038	0.731 ± 0.065	**0.913 ± 0.046**	0.827 ± 0.134
Serology (core)	Stacking	0.693 ± 0.075	0.493 ± 0.073	0.693 ± 0.046	0.586 ± 0.040
Serology (extended)	Random forest/Elastic net	0.659 ± 0.051	0.532 ± 0.067	0.677 ± 0.043	0.504 ± 0.067
Combined (endoscopy + serology)	Random forest	**0.878 ± 0.051**	0.749 ± 0.040	0.907 ± 0.065	0.886 ± 0.033

Values are mean ± SD across stratified 5-fold cross-validation.

AUC, area under the receiver operating characteristic curve; ME-NBI, magnifying endoscopy with narrow-band imaging; RF, random forest; WLE, white-light endoscopy.

Bold values indicate the best-performing model for each feature set and task.

### Feature importance and SHAP interpretability

Loss of gastric folds ranked first in every endoscopy-based model. Mucosal thinning and visible submucosal vessels consistently entered the top five; sticky adherent dense mucus and swelling of the areae gastricae also contributed. In the WLE+ME-NBI binary model, CO grade and Barrett esophagus grade ranked second and third. Among serum indicators, G-17 and PGI alternated in the top two ranks across the three-class models; PGI moved to first place in the binary serology models, and the PGI/PGII ratio was strongly informative in the combined model. PCA titer, folate, and vitamin B12 had modest discriminative value.

**Figure 3:**
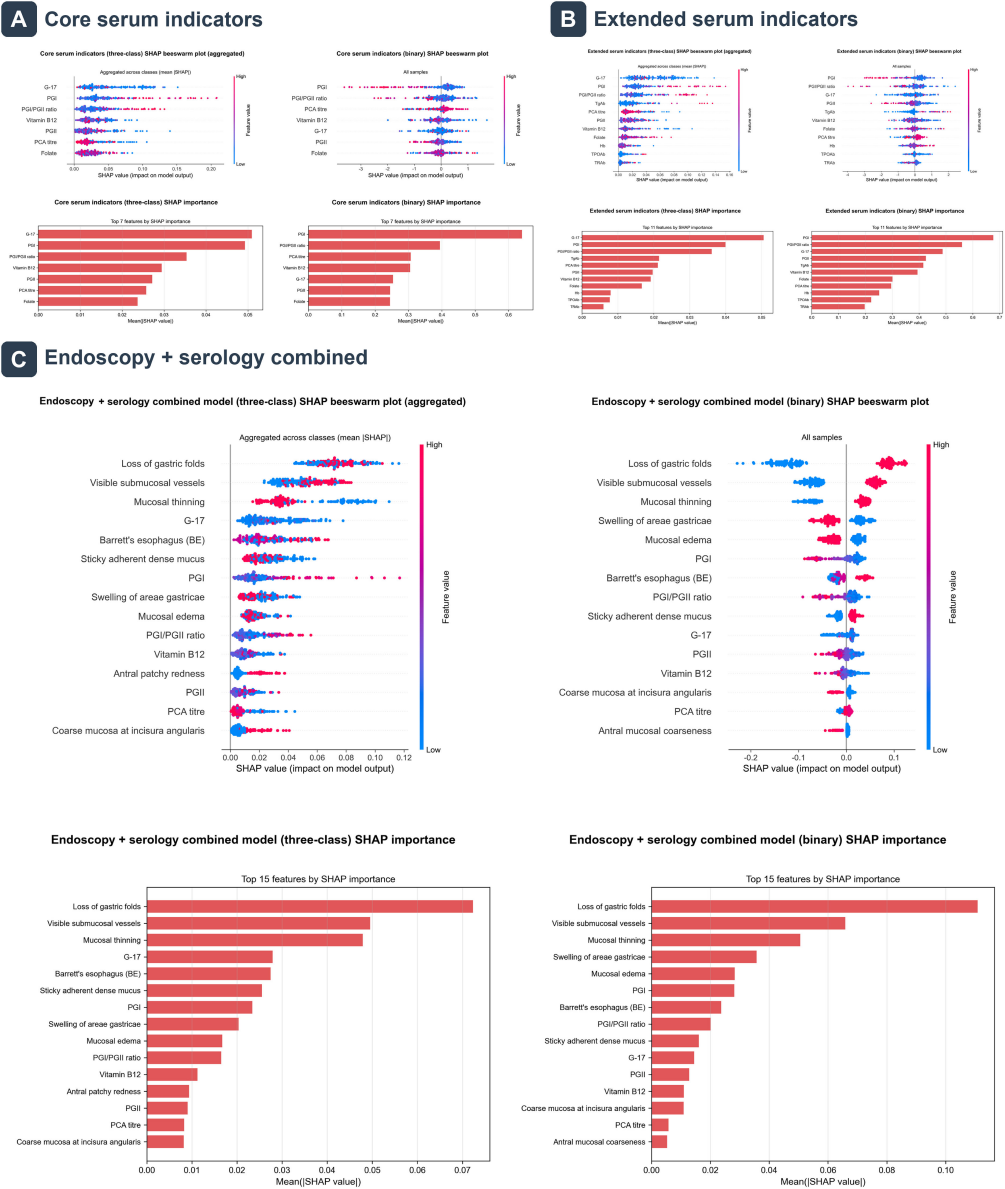
SHAP analyses for serology-based and combined feature sets. **(A)** Core serum panel. **(B)** Extended serum panel. **(C)** Combined endoscopy + serology — the best three-class model overall. Each sub-panel contains the three-class beeswarm plot, the binary beeswarm plot, and the corresponding mean(|SHAP|) bar plots. Atrophy-related features and low pepsinogen I push predictions toward advanced disease; inflammation-related features push toward early disease.

SHAP analysis (**Figure 3**) showed that the presence of loss of gastric folds, mucosal thinning, visible submucosal vessels, CSA, absent CO, low PGI/PGI–PGII ratio, and long-segment Barrett esophagus pushed predictions toward advanced disease, whereas mucosal edema, swelling of the areae gastricae, MCE swelling, high PGI, and low G-17 pushed predictions toward early disease. Class-specific beeswarm plots showed that Stage 1 was strongly associated with inflammation features and the absence of atrophic features; Stage 3 was strongly associated with atrophic features and long-segment Barrett esophagus; and Stage 2 showed a diffuse SHAP distribution with small-magnitude contributions in both directions, providing an interpretability-level explanation for its relatively lower classification accuracy.

### Multicollinearity

In the combined set (31 variables), VIFs were 8.89 for PGI and 6.89 for the PGI/PGII ratio—mechanistic, as the ratio derives from PGI—with all other VIFs <5.0. Pearson |r|>0.7 occurred only between PGI and the PGI/PGII ratio. All features were retained because the best combined model (random forest) is robust to collinear inputs.

## Discussion

To our knowledge, this is the first machine-learning study to address within-AIG stage prediction. Across five feature sets and seven algorithms in 203 integrated-criteria patients, endoscopic features substantially outperformed serological features, combining the two modalities yielded the best three-class performance (combined macro-AUC 0.878), and the binary “advanced vs earlier” distinction was achievable from endoscopy alone (WLE+ME-NBI AUC 0.913). The cohort is among the larger Chinese AIG series to date, and nested cross-validation with leakage-controlled SMOTE provides a conservative estimate of generalization. We contextualize the principal findings below.

### Stage 2 as a transitional, intervention-relevant window

Stage 2 was consistently the hardest class to identify—not a methodological artifact, but a biological consequence. Its phenotype combines residual early inflammation (mucosal edema, swelling of the areae gastricae) with emerging atrophy (sticky adherent mucus, CSA, partial loss of folds). SHAP distributions for Stage 2 were diffuse, with small-magnitude contributions in both directions. Mechanistically, Stage 2 corresponds to peak G-17 elevation (median 34.8 pmol/L) and maximal G-cell compensation, with the most active glandular metaplasia (17). Pseudopyloric and intestinal metaplasia are still progressing and parietal-cell injury is ongoing (18); this is the disease window in which interrupting autoimmune injury could plausibly retard progression, and any future trial of immunomodulatory therapy should prioritize Stage 2.

### Loss of gastric folds: a near-linear surrogate of ROM

Across every endoscopy-based model, loss of gastric folds was the dominant predictor; mucosal thinning and visible submucosal vessels consistently entered the top five. Mechanistically this is expected: when oxyntic glands are lost the corpus folds lose their turgor, and once ROM falls below ~10% the folds disappear entirely. The three features therefore form a compact, reproducible endoscopic surrogate for advanced AIG that is more readily standardized than visual ROM estimation. Sticky adherent dense mucus, observed in 67.5% of Stage 3 patients and attributed to overgrowth of non–*H. pylori* urease-producing bacteria in the achlorhydric stomach (9), provides a further visual cue. Swelling of the areae gastricae and MCE swelling—classical inflammation signatures—declined with stage and pushed SHAP toward early disease, consistent with the deep-lamina-propria lymphocytic infiltration characteristic of early AIG (19, 20).

### Barrett esophagus link

The most novel finding is the 66.8% prevalence of Barrett esophagus in our cohort—an order of magnitude higher than the ~7% in unselected gastroesophageal reflux disease (21)—with a clear shift from short-segment to long-segment Barrett esophagus across stages (long-segment 13.4% at Stage 1 vs 43.0% at Stage 3). The classical reflux model cannot easily explain this, because AIG progressively eliminates gastric acid (22). Two acid-independent mechanisms are plausible. First, weakened mucosal barrier function in the atrophic stomach allows duodenal bile reflux into the esophagus, producing direct chemical injury (23). Second, sustained hypergastrinemia stimulates CCK2R-positive cardiac progenitor cells to give rise to Barrett-like columnar epithelium (24). AIG combines achlorhydria with hypergastrinemia and provides an unusually favorable substrate for Barrett progression. The practical implication is that explicit esophageal evaluation should be a routine component of endoscopic follow-up in AIG.

### Endoscopy versus serology

Serology-only models reached only AUC ~0.69 in either task (26). PGI and G-17 did not differ significantly between Stages 2 and 3, indicating an intrinsic ceiling on serological staging (25). Adding thyroid autoantibodies and hemoglobin did not improve performance and may have added noise. Combining endoscopy with the core serum panel improved the three-class task (0.878 vs 0.853) but not the binary task beyond endoscopy alone, suggesting that fine-grained staging benefits modestly from serology as an adjunct while binary advanced-disease identification can rely on endoscopy.

### Comparison with prior work

Image-based deep-learning systems for AIG diagnosis [e.g., CorpusNet-AIG (11) and multi-site fusion models (12)] achieve diagnostic AUCs above 0.97 but address AIG-vs-non-AIG classification rather than within-AIG staging; the present work complements rather than competes with that literature. Our SHAP-based interpretability quantifies the direction and magnitude of each clinical feature’s contribution, complementing the pixel-level Grad-CAM saliency used in image-based AI. Our findings should be interpreted within the broader clinical and pathological spectrum of autoimmune gastritis and chronic gastritis (27, 28).

### Limitations

This is a single-center, retrospective study; multicenter external validation is needed. ROM area is still observer-judged near stage cut-offs, and an objective, automated quantification method is a natural next step. We did not perform feature-by-feature histologic correlation; prospective biopsy-anchored cohorts will strengthen pathologic interpretation. Larger Stage 2 cohorts may improve fine-grained discrimination.

## Conclusions

In 203 integrated-criteria AIG patients, machine learning provided a feasible, interpretable framework for staging within-disease severity. Endoscopic features—dominated by loss of gastric folds, mucosal thinning, and visible submucosal vessels on WLE, and by crypt-opening grade and cast-off skin appearance on ME-NBI—outperformed serological indicators. Combining endoscopy with core serum markers yielded the best three-class staging (combined macro-AUC 0.878), while endoscopy alone was sufficient for binary advanced-disease identification (WLE+ME-NBI AUC 0.913, sensitivity >0.80). Stage 2 emerged as a transitional, intervention-relevant window in which autoimmune injury is still active and glandular metaplasia is at its peak. The 66.8% prevalence of Barrett esophagus, with long-segment disease enriched 3.3-fold in advanced AIG, supports routine, explicit esophageal evaluation during AIG surveillance. After multicenter external validation, this multi-feature atlas and machine-learning workflow may inform precision surveillance and individualized clinical management of AIG, particularly for the timely initiation of neoplastic surveillance in advanced disease.

## Data Availability

The raw data supporting the conclusions of this article will be made available by the authors, without undue reservation.
